# Point-of-Care Ultrasound Overview and Curriculum Implementation in Internal Medicine Residency Training Programs in the United States

**DOI:** 10.7759/cureus.42997

**Published:** 2023-08-05

**Authors:** Solomon O Badejoko, Nso Nso, Cyrus Buhari, Omar Amr, John P Erwin

**Affiliations:** 1 Internal Medicine, St. Joseph’s Medical Center (Dignity Health), Stockton, USA; 2 Internal Medicine/Cardiology, NorthShore University HealthSystem/University of Chicago Pritzker School of Medicine, Illinois, USA; 3 Cardiology, St. Joseph’s Medical Center (Dignity Health), Stockton, USA; 4 Emergency Medicine (Ultrasound), St. Joseph’s Medical Center (Dignity Health), Stockton, USA; 5 Medicine/Cardiology, NorthShore University HealthSystem/University of Chicago Pritzker School of Medicine, Illinois, USA

**Keywords:** point of care ultrasonography, non-invasive imaging modalities, internal medicine training, innovation in medical education, curriculum implementation, point of care echocardiography, point-of-care-ultrasound

## Abstract

Point-of-care ultrasonography (POCUS) augments physical examination and expedites diagnostic care and clinical decision-making. The use of POCUS in internal medicine (IM) appears inconsistent despite its commendable benefits. It is not fully incorporated into the IM residency core competency skills or academic curriculum. This narrative literature review explores the benefits of POCUS and evaluates the need for an IM-focused POCUS curriculum. The obstacles and a proposed curriculum are also described.

## Introduction and background

Point-of-care ultrasonography (POCUS), also called bedside clinical ultrasound, challenges conventional diagnostic and clinical care by enabling faster diagnostic decision-making. Through real-time dynamic visualization, it provides a more rapid assessment of pathophysiologic processes in the hands of a clinician who is also well aware of the patient’s history and other physical examination findings. Some have hypothesized that POCUS may replace the stethoscope eventually [1-3].

Structured training on POCUS, however, is scarce in most internal medicine (IM) residency programs in the United States [4,5]. There are inadequate large interventional IM-focused POCUS studies. In addition, POCUS is presently not a mandatory core competency in IM residency training. This literature review aims to identify the benefits, limitations, barriers, and processes of incorporating POCUS into the IM residency curriculum in the United States.

## Review

General benefits of POCUS

POCUS serves as a teachable, convenient, cost-effective, ionizing radiation-free, and non-invasive adjunct to conventional physical examination [6]. After a brief 18-hour POCUS training, medical trainees using POCUS-aided examination had a 26% higher overall diagnostic accuracy, compared to board-certified cardiologists who performed clinical physical examinations alone. These trainees had a 31% higher sensitivity for valvular lesions, 49% higher sensitivity for diastolic murmur etiology, and 14% higher sensitivity for non-valvular pathologies [6]. In comparison to chest radiography (CXR), POCUS is 42% more sensitive for pneumothorax, 15% more sensitive for pulmonary edema, and 18% more sensitive for pneumonia, all with a comparative specificity of at least 90% [4,7-9]. Compared to cardiologists, a meta-analysis showed at least 80% sensitivity and specificity for ejection fraction (EF) estimation when performed by primary care internists in outpatient settings [4,7]. In inpatient settings, POCUS is additionally used to evaluate the etiology of non-specific acute dyspnea and undifferentiated hypotension [10].

**Figure 1:**
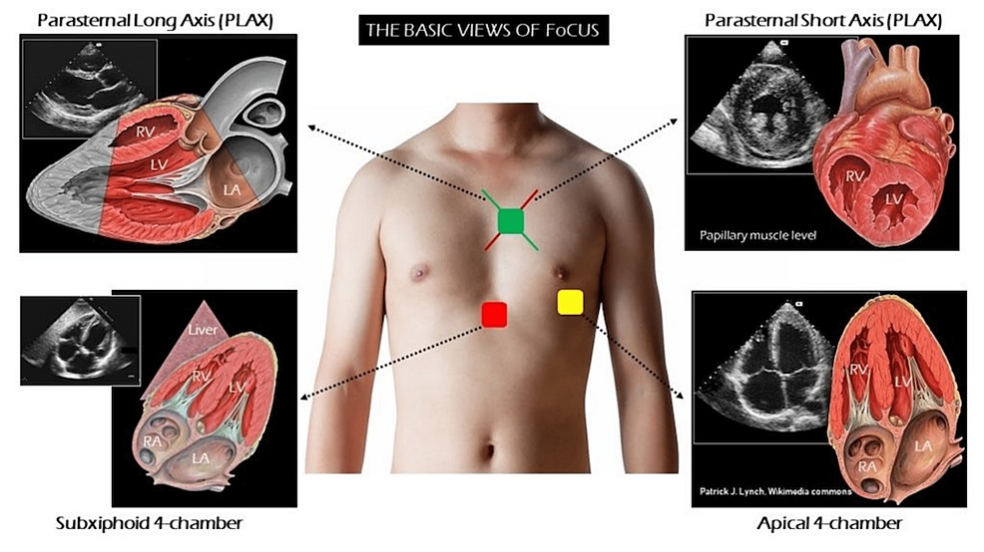
The common views of POCUS trans-thoracic echocardiography Source: Renal Fellow Network [14]. Reprinted with permission. POCUS: Point-of-Care Ultrasound

**Figure 2:**
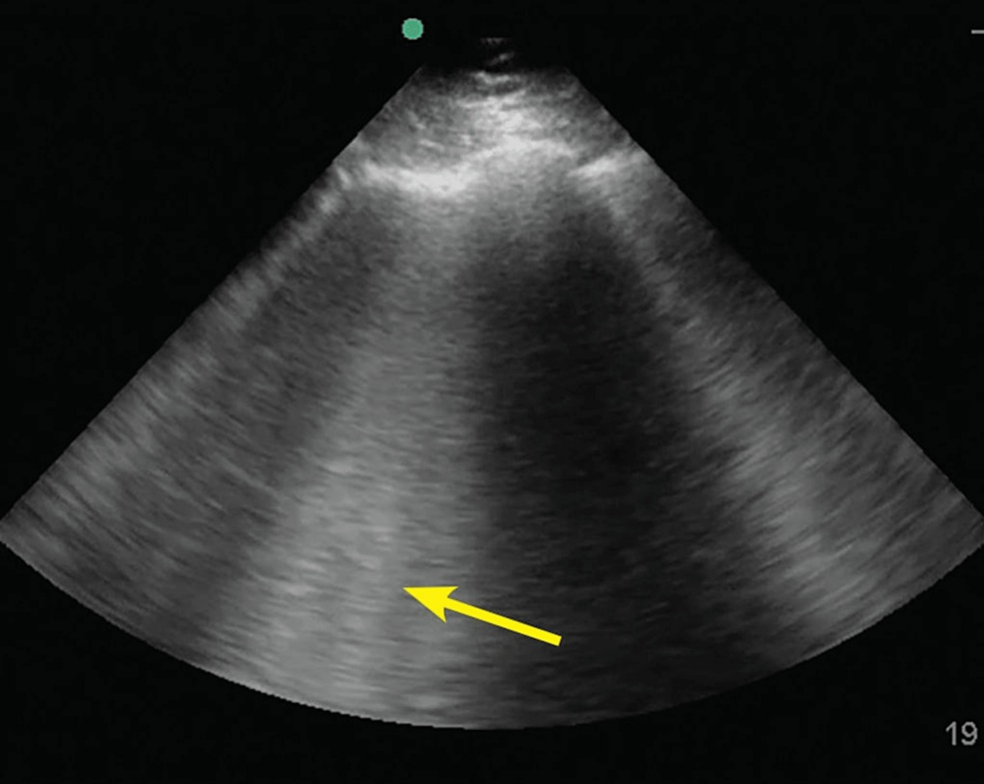
Multiple B-lines in pulmonary edema Source: Thind et al., 2021 [15]. Reprinted with permission.

The American College of Physicians and the American College of Cardiology recommend the use of POCUS for non-specific dyspnea. It increases diagnostic accuracy by 32% when coupled with standard diagnostic tests [10-12]. For equivocal modified Well’s criteria score and D-dimer level in the setting of suspected pulmonary embolism, a multi-organ POCUS had at least 85% sensitivity and specificity (higher than CT angiography [13,14]. For example, a multi-organ POCUS comprises POCUS echocardiography (echo) as shown in Figure 1, vascular ultrasound, and lung ultrasound as shown in Figure 2. POCUS increased clinical pretest probability further reduces the need for CT scans [13-15].

**Figure 3:**
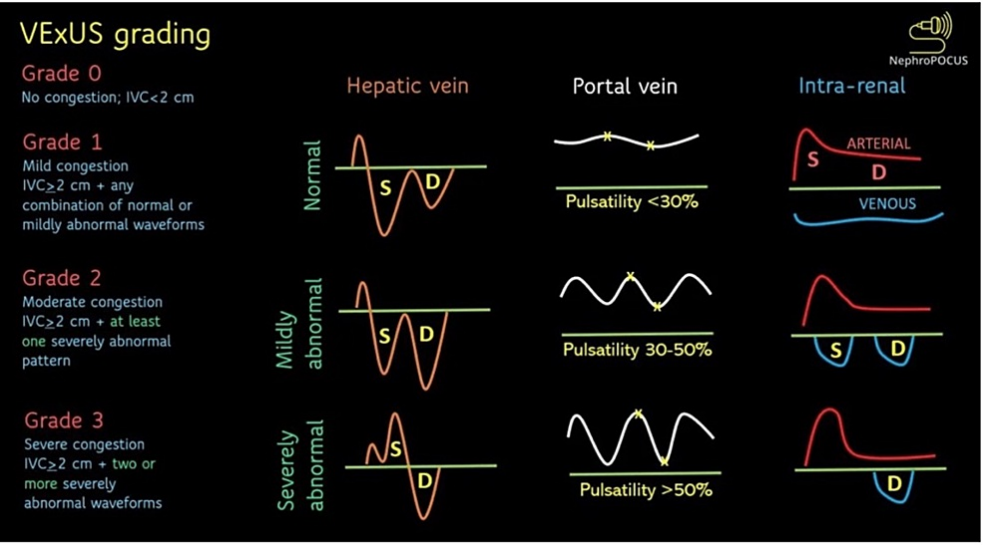
Vascular Doppler ultrasound of abdominal organs Source: Koratala and Reisinger, 2022 [21]. Reprinted with permission. VExUS: venous excess ultrasound; IVC: inferior vena cava

POCUS also guides the safe peripheral intravenous catheter placement for patients with inconspicuous peripheral veins. This avoids multiple failed attempts, improves patient experience, and improves trust in care [16]. There was a 14% improved confidence in POCUS-guided procedures noticed in a similarly large IM residency program [17]. It reduces unnecessary further testing by 63% [18]. Its cost-saving benefits, decreased ICU length of stay, shorter duration of mechanical ventilator dependence, and reduction in the need for pressors have been described [8,18]. Assessment of adequate diuresis in patients with fluid congestion may be augmented with vascular Doppler ultrasound of abdominal organs as shown in Figure 3 [8,19-21]. Its versatility in primary care, potentially makes it an effective screening tool for deep venous thrombosis (DVT) and life-threatening conditions such as aortic aneurysm [17].

Need for POCUS incorporation in IM curriculum

Bedside auscultative and observatory examination skills have been declining in most Western countries in recent decades. To provide an example of modern Western practice, a dyspneic patient with presumed congestive heart failure (CHF) exacerbation routinely receives a chest radiograph, brain natriuretic peptide (BNP) lab, and complete transthoracic echocardiography (TTE). If there is a high-risk probability of pulmonary embolism, CT angiography of the chest is often ordered. The escalated imaging procedures create a time delay which has both patient safety and significant cost implications. Jugular venous distension, LV S3 heart sound, and other clinical signs are inconsistently identified [9,21]. Factors contributing to the quality decline of auscultatory and examination skills among trainees include a shorter time spent at the bedside, poor inter-observer agreement, inadequate training capacity, and inconsistent bedside demonstration. Others include dependence on expensive radiographic alternatives, rapid insurance-driven turnover for patients, and the rise of ‘defensive medicine’ in the United States’ litigious system [2,3].

With this in mind, clinical decision-making can be expedited with POCUS echocardiography. This screens for reduced EF, severe pericardial effusion/ tamponade, right ventricular (RV) strain, regional wall motion abnormalities, valvular abnormalities, etc. Bedside lung ultrasound screens for pulmonary edema, bilateral pleura sliding, and pleural effusion. With possible pulmonary embolism in mind, a quick DVT ultrasound could also be performed [4,7,14].

Obstacles to POCUS IM residency curriculum implementation

Over 90% of IM residents in a community hospital identified a lack of a formal POCUS curriculum as a major impediment to obtaining the necessary skills for modern practice and training [18,22]. There are perceived high costs of ultrasound equipment purchases. Paradoxically, POCUS use is associated with significantly reduced healthcare costs (about $20,000 per critically ill patient requiring an ICU stay) [8]. This cost-saving benefit is associated with a trend toward decreased morbidity, vasopressor use, and mechanical ventilation dependence [8,23-25]. A reliable reimbursement system for performed POCUS is still emerging. Presently, physicians may only code for a higher level of billing complexity for POCUS as an ‘extended physical examination’ [26-29]. It is critical that the clinician adequately archive their images and POCUS-guided procedures. Many health systems don’t have a secure digital archiving system (PACS) for POCUS images. When they do, most POCUS operators inconsistently store their images. This makes it difficult to compare their findings with those of radiologists and other ultrasound experts [26-28]. There are also challenges of inaccurate reads, stemming from less exposure, limited supervised faculty expertise, faculty preference for other imaging modalities, perception of limited need, push-back from other competing specialties [18], and other logistic impediments [18,22]. Inaccurate reads may potentially derail medical decision-making and compromise patient safety. At present, it is unclear if POCUS-based decisions are legally defendable. The use of POCUS for all patients seen in busy clinics or inpatient units may be unrealistic. However, it might be practical to use it to further asses the sicker patients. The lack of sustained support for a longitudinal program weakens the program’s chances. Also, POCUS-trained faculty may not always be available to review the resident-acquired images. Although single training sessions are a good start, IM residents in programs without a structured curriculum experience a significant comparative decline (29-50%) in skills such as identification of pleural effusion and ascites [29]. This reinforces the need for repetition and frequent usage of knowledge retention [30].

The accuracy of POCUS is dependent on the operator’s skill and judgment. Unexpected incidental findings often lead to more unnecessary testing escalation [14,18]. This may be mitigated by first comparing residents’ incidental findings with a POCUS-certified attending. The inter-reader agreement needs to be studied in a controlled IM-focused prospective RCT design. Lastly, while some have argued that POCUS use may not provide significant survival benefits [14,19], POCUS provides reliable physical examination augmentation [30,31].

A model IM POCUS curriculum implementation at a community hospital

In a short cross-sectional needs assessment survey at our community hospital (St. Joseph’s Medical Center, Stockton, California), 70% of participating trainees (residents) had never performed the most basic POCUS modules before. A pre-intervention assessment survey was obtained, using a validated questionnaire [23], as part of a quality improvement initiative. Seventy percent of the participating residents were uncomfortable performing POCUS echo or lung examination in the setting of trauma (p-value < 0.001), or facilitating clinical decision-making, while 75% of resident participants felt that a multi-modal teaching approach will improve their management of patients with non-specific dyspnea and undifferentiated hypotension.

**Figure 4:**
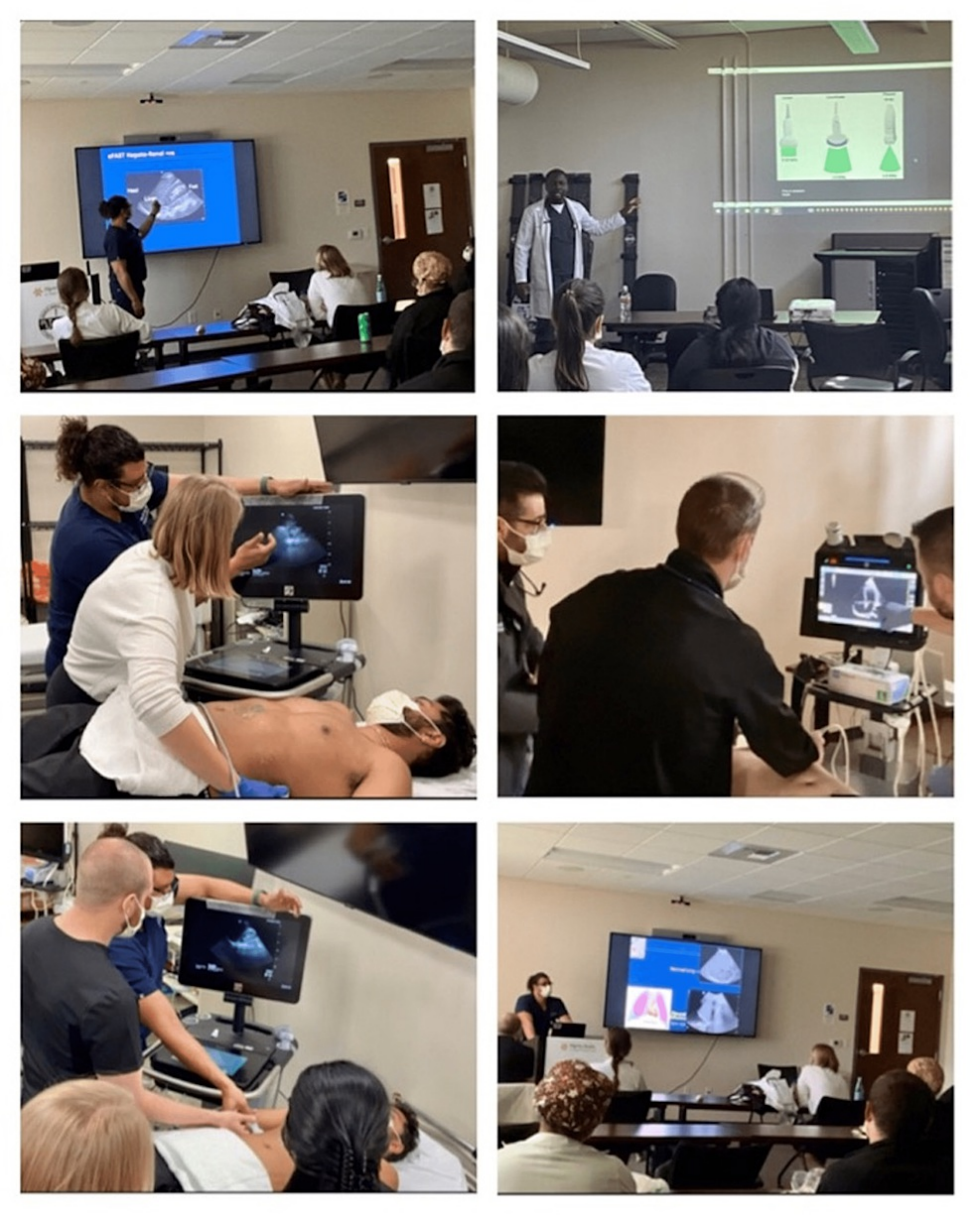
POCUS didactic session images for internal medicine residents at SJMC SJMC: St. Joseph’s Medical Center, Stockton, California. Permission was obtained from the participants in the group photographs.

These findings inspired the design and implementation of our institution's first IM resident-driven peer-to-peer POCUS curriculum. Six modules (didactic lectures) were taught over two months. These were followed by live model simulation sessions (Figure 4). The modules included basic ultrasound physics and ultrasound machine familiarization. It also included basic echocardiography (parasternal long, parasternal short, apical four-chamber, and the sub-xiphoid views), with a focus on ejection fraction estimation, wall motion abnormalities, and pericardial effusion. The lung ultrasound module screened for pneumothorax, pulmonary edema, pleural effusion, and pneumonia. The core vascular module screened for a DVT, and an abdominal aorta aneurysm. The extended focused assessment with sonography in trauma (e-FAST) module screened for hemoperitoneum, hemopericardium, hemothorax, and a post-void bladder residual volume. We procured two cart-based and six handheld portable ultrasound devices with a high-fidelity resolution, as well as an affordable upfront and maintenance cost. These machines are connected to an efficient Health Insurance Portability and Accountability Act (HIPAA)-compliant and cyber-secure archiving system.

Future plans and recommendations

Future plans include the POCUS incorporation at interns' boot camp during the onboarding orientation schedule. A longitudinal approach with monthly ultrasound-based morning reports and afternoon ultrasound rounds is planned. POCUS-certified instructors will gauge the competence and accuracy of residents’ skills during real scanning sessions. Approximately 25-50 mentored scans are considered sufficient to evaluate for pericardial effusions, LV contractility, etc. However, a periodic refresher course is needed to maintain the knowledge and skills. Ultrasound electives are necessary to train POCUS ‘champions’, who can sustain the peer-to-peer teaching and facilitate POCUS noon conferences (in conjunction with the emergency medicine, critical care, and simulation departments). Advanced POCUS modules may be added subsequently e.g., gall bladder, peripheral arterial disease Doppler screening, fluid status (with venous excess ultrasound (VExUS)) [20], valvular heart disease, and raised intracranial pressure estimation (with ocular POCUS). Interested IM attendings will complete a pre-requisite number of POCUS exams needed for credentialing [30,31].

## Conclusions

POCUS is an efficient augmentation of the physical examination skillset that improves diagnostic care, enhances patient safety, and could potentially drive innovation for our healthcare systems, especially in resource-limited settings. IM training programs need to incorporate this structured longitudinal POCUS curriculum as a core competency.
